# Mimicking the Nucleosomal Context in Peptide-Based Binders of a H3K36me Reader Increases Binding Affinity While Altering the Binding Mode

**DOI:** 10.3390/molecules25214951

**Published:** 2020-10-26

**Authors:** Velten Horn, Seino A. K. Jongkees, Hugo van Ingen

**Affiliations:** 1 Macromolecular Biochemistry, Leiden Institute of Chemistry, Leiden University, P.O. Box 9502 Leiden, The Netherlands; v.horn@lic.leidenuniv.nl; 2 Chemical Biology and Drug Discovery Group, Utrecht University, P.O. Box 80082 Utrecht, The Netherlands; s.a.k.jongkees@uu.nl; 3 NMR Group, Bijvoet Centre for Biomolecular Research, Utrecht University, Padualaan 8, 3584 CH Utrecht, The Netherlands

**Keywords:** epigenetic drugs, histone modifications, reader proteins, methyllysine, NMR, PSIP1, PWWP, H3K36

## Abstract

Targeting of proteins in the histone modification machinery has emerged as a promising new direction to fight disease. The search for compounds that inhibit proteins that readout histone modification has led to several new epigenetic drugs, mostly for proteins involved in recognition of acetylated lysines. However, this approach proved to be a challenging task for methyllysine readers, which typically feature shallow binding pockets. Moreover, reader proteins of trimethyllysine K36 on the histone H3 (H3K36me3) not only bind the methyllysine but also the nucleosomal DNA. Here, we sought to find peptide-based binders of H3K36me3 reader PSIP1, which relies on DNA interactions to tightly bind H3K36me3 modified nucleosomes. We designed several peptides that mimic the nucleosomal context of H3K36me3 recognition by including negatively charged Glu-rich regions. Using a detailed NMR analysis, we find that addition of negative charges boosts binding affinity up to 50-fold while decreasing binding to the trimethyllysine binding pocket. Since screening and selection of compounds for reader domains is typically based solely on affinity measurements due to their lack of enzymatic activity, our case highlights the need to carefully control for the binding mode, in particular for the challenging case of H3K36me3 readers.

## 1. Introduction

Post-translational modifications of histone proteins, loosely referred to as epigenetic modifications, play a key role in regulating nuclear processes such as gene expression and DNA repair, replication, or transcription [1,2]. Deregulation of the installation, recognition or removal of these modifications, such as phosphorylation and lysine ubiquitination, methylation or acetylation, are intimately associated with a wide range of pathologies. As such the proteins that write, read or remove histone modifications have emerged as promising therapeutic targets, resulting in a growing number of small-molecule and peptide-based epigenetic drugs [3,4,5,6]. 

Particularly promising are inhibitors targeting reader proteins of specific histone modifications, allowing for selective interference with the biological pathway regulated by the targeted reader protein without general inhibition of the modification and leaving additional functions of the protein untouched [7,8,9]. Most progress has been made in the development of inhibitors that target bromo domain readers, which recognize acetylated lysines [10]. Recently, potent inhibitors of lysine methylation reader have been developed [11,12,13,14], but in general, the number of promising inhibitors for this class of reader proteins is significantly lower [15,16]. Methyllysine recognition is driven by cation–π interactions between several aromatic residues forming an aromatic cage and the methylated lysine head-group [17,18]. While in some cases this cage forms a deep cavity, as for monomethylated lysine binding, in many other cases, and in particular for trimethyllysine, the cage forms a shallow pocket [19]. The lack of a pronounced binding cavity makes trimethyllysine readers an intrinsically challenging target for small-molecule inhibition. Peptide-based inhibitors offer an attractive approach to engineer increased binding affinity and specificity in such cases [20,21]. Their development may benefit from studying the native binding mode of reader proteins and has led to a promising inhibitors [22,23,24]. In many cases the native chromatin binding mode of reader proteins also involves DNA or RNA binding through positively charged patches on the protein surface [25]. This offers in potential an additional binding interface that can be exploited in the design of inhibitors. So far, inhibitors have been developed that block binding of the reader to the targeted modification but allosterically enhance DNA/RNA binding [24] and that bind preferably to RNA-bound forms of the reader protein [26].

**Figure 1:**
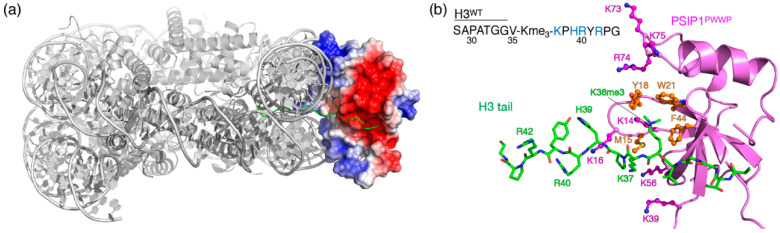
The synergetic nature of PSIP1^PWWP^ binding to nucleosomes. (**a**) Structure of PSIP1^PWWP^ bound to nucleosomes with trimethylated H3 tail residue K36 (H3K36me3), illustrating the electrostatic interactions of positively charged surface residues on the PWWP domain with both gyres of nucleosomal DNA [36,38]. The H3 tail is shown in green. (**b**) Details of the PSIP1-H3 tail interaction, with the aromatic cage (orange) and DNA binding (magenta) residues shown as sticks. The native H3 tail peptide (H3^WT^) sequence is annotated with positively d charged residues highlighted.

Here, we explore the potential of peptide-based inhibitors for reader proteins of trimethylated lysine 36 in histone H3 (H3K36me3), a modification that is found in the gene body of actively transcribed genes [27]. Unlike most modifications that occur on the flexible N-terminal tails that protrude from the nucleosome core, H3K36 sits very close to the nucleosomal DNA, creating a distinct supramolecular context. One of the H3K36me3 reader proteins is transcriptional co-activator PSIP1 (also known as LEDGF) [28,29,30], which has been linked to HIV integration, autoimmune disease and cancer [31,32,33,34,35]. We and others have shown that recognition of H3K36me3 by the PWWP domain of PSIP1 is driven by non-specific interactions with the nucleosomal DNA around the modification (see Figure 1) [36,37,38]. Such synergistic DNA and trimethyllysine binding is observed also for other K36me readers [39] and is thought to be a general feature of these proteins. These characteristics however pose a significant challenge for design of peptide-based inhibitors as the binding of H3K36me3 peptides is typically very weak with dissociation constants (*K*_D_) in the millimolar range [36,40,41,42,43]. We thus aimed to incorporate the binding synergy present in the native nucleosomal context into a peptide model. We designed a series of linear and branched peptides that include both the trimethyllysine and negative charges to mimic the nucleosomal DNA. With detailed interaction studies using NMR spectroscopy, we show that by increasing the negative charge of the peptide, binding affinity can be boosted up to ~50-fold. Peptides with increased electrostatic interaction to the protein had reduced occupancy of the aromatic cage. This indicates an incompatibility between the two binding modes in our peptide models, highlighting the need to carefully optimize the fit of both binding epitopes to the protein surface.

## 2. Results

### 2.1. Electrostatic Repulsion Decreases the Affinity of H3K36me3 Peptides for PSIP1^PWWP^

We showed previously that the PSIP1-PWWP domain (PSIP1^PWWP^) binds a H3K36me3 peptide with a *K*_D_ of ~17 mM, while in the nucleosomal context, the interaction is several orders of magnitude tighter (*K*_D_ in the low micromolar range) [30]. Before designing nucleosome-mimicking peptides, we first examined the role of three positively charged residues in the H3 tail, H39, R40 and R42, that are in close proximity to the K36 modification site (Figure 1). In the nucleosome, these are located in-between the two DNA gyres and thus their positive charges are essentially masked. However, when studying the binding of PSIP1^PWWP^ to a H3 tail peptide, these charges will no longer be masked, but fully exposed to the protein. We therefore hypothesized that the non-native electrostatic nature of a peptide-based H3 tail model peptide leads to unfavorable repulsion with positive surface residues of PSIP1^PWWP^. 

To dissect the influence of these residues, we mutated H39, R40 and R42 to alanine, leaving K37 untouched to avoid altering the local structure around K36me3, and studied the binding of this partly neutralized H3 tail peptide (H3^A3^) to the PSIP1^PWWP^ domain using NMR spectroscopy. NMR is particularly well suited in this case because it allows the study of low affinity interactions as well as detailed insights into the binding mode of the peptide.

**Figure 2:**
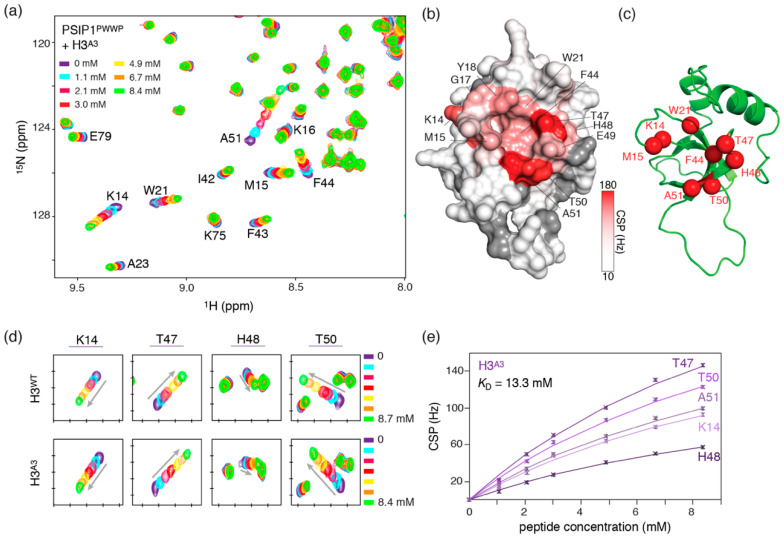
Removal of positive charges in the H3 tail peptide modestly increases PSIP1^PWWP^ binding. (**a**) Zoom on overlaid ^15^N-HSQC spectra of PSIP1^PWWP^ with increasing amounts of H3^A3^ added, showing clear and specific chemical shift perturbations (CSPs). Color coding indicated. (**b**) Surface plot of PSIP1^PWWP^ with color coding of CSPs (observed at 8.4 mM peptide added), showing largest CSPs in and around the aromatic cage. (**c**) Location of residues used for reporting on aromatic cage binding. (**d**) Zoom on the observed CSPs for aromatic cage reporter residues in both H3^WT^ and H3^A3^ titrations, showing highly similar peak trajectories indicating a similar binding mode. Tic marks are placed each 0.1/0.5 ppm in the ^1^H/^15^N dimension. (**e**) NMR-derived binding curves with best fits (solid lines) for selected residues together with best-fit value for the *K*_D_ using a 1:1 binding model global fit of all residues with significant CSP. The plotted binding curves were derived from the CSPs in the ^1^H dimension.

Addition of the mutant H3-tail peptide to ^15^N-labelled PSIP1^PWWP^ domain resulted in clear and specific chemical shift perturbations (CSPs). We observed pronounced CSPs for several residues including T47, T50, A51 that are part of the β4-strand and known to form a beta-sheet with H3 tail residues 34–36 (Figure 2a–c). Comparison with titration data of the native H3 tail peptide (H3^WT^) [36] shows that the affected residues for both peptides are clustered in two main binding sequences, K14–A23 and F44-A51, resulting in the same mapped binding site in and around the aromatic cage (Figure 2b and Figure S1). Furthermore, comparison of the H3^WT^ and H3^A3^ titrations shows that the peak trajectories have nearly identical directions, indicating comparable bound-state chemical shifts and thus a comparable bound-state conformation for the mutant complex (Figure 2d). A global fit of the complete titration data (up to ~8 mM peptide) yields a best-fit *K*_D_ value of 13.3 mM for H3^A3^ (Figure 2e). The low affinity hampers precise determination of the *K*_D_, which is reflected in the rather wide 95% probability limits for the *K*_D_, with lower limit of 9.3 and upper limit of 21.1 mM. These values are overall lower than the *K*_D_ of 17.3 mM (13.9–22.1 mM 95% probability limits) for the H3^WT^ peptide. Thus, our data indicate that the H3^A3^ peptide binds in a native binding mode to PSIP1^PWWP^ with a modestly increased affinity. We conclude that charge repulsion between H39, R40, R42 and the positive PWWP surface contributes modestly to the low affinity of PSIP1^PWWP^ towards H3 tail peptides and that additional attractive interactions are needed to boost the affinity further.

### 2.2. Introduction of Negative Charges Enhances Peptide Binding to PSIP1^PWWP^

**Figure 3:**
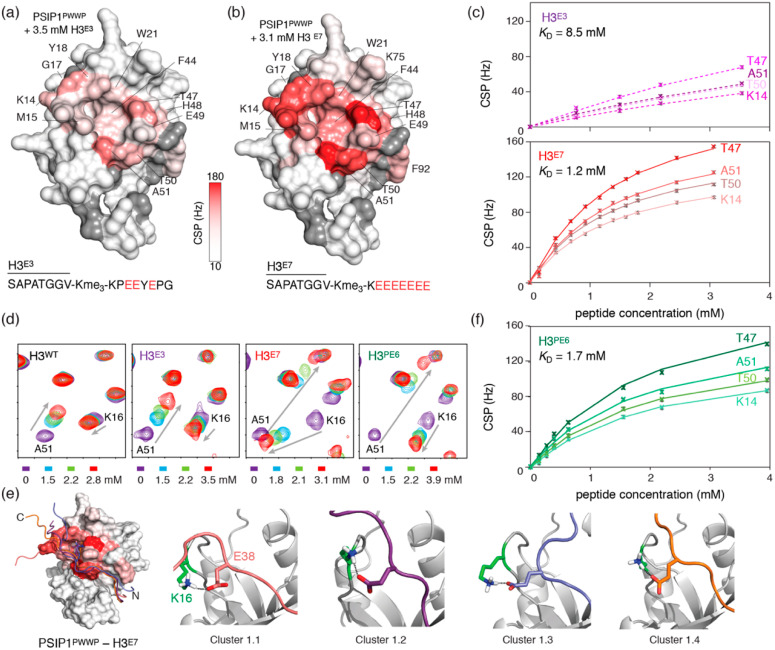
Glu-rich H3 peptides have increased affinity for PSIP1^PWWP^. (**a**,**b**) Surface plot of PSIP1^PWWP^ with color coding of CSPs upon binding of H3^E3^ (**a**) and H3^E7^ (**b**) at the indicated peptide concentration, showing largest changes around the aromatic cage region. (**c**) NMR-derived binding curves for H3^E3^ (top) and H3^E7^ (bottom) with best fits (solid lines) for selected residues together with best-fit value for the *K*_D_ using a 1:1 binding model global fit of all residues with significant CSP. Fits for H3^E3^ are based on titration up to 8.28 mM peptide; full data are shown in Figure S2. (**d**) Zoom on peak trajectories for A51 and K16 in the H3^WT^, H3^E3^, H3^E7^ and H3^PE6^ titrations, showing significantly increased CSP for K16 in the H3^E7^ titration. Tic marks are placed each 0.05/0.5 ppm in the ^1^H/^15^N dimension. (**e**) Structural models of H3^E7^ bound to PSIP1^PWWP^, showing top four structures overlaid (left) and zoomed in on a conserved hydrogen bond between residues K16 (PSIP1^PWWP^) and E38 (H3^E7^) (right). (**f**) NMR-derived binding curves for H3^PE6^ with best fits (solid lines) for selected residues together with best-fit value for the *K*_D_ using a 1:1 binding model global fit of all residues with significant CSP. Fits are based on titration up to 5.5 mM peptide; full data are shown in Figure S2. The plotted binding curves in panels c and f were derived from the CSPs in the ^1^H dimension.

We next designed two H3 tail peptide models that incorporate negatively charged residues to mimic the additional attractive electrostatic forces generated by the DNA in the nucleosomal context. In the first sequence, the three positively charged residues H39, R40 and R42 were mutated to glutamate (denoted as H3^E3^). Since in the native binding mode the C-terminal part of the H3 tail is orientated towards the DNA-binding surface of PSIP1, we designed a second sequence, in which all seven residues on the C-terminal side of Kme3 are substituted by glutamate to create a highly negatively charged H3 tail model (H3^E7^). By comparing the effects of these two Glu-rich peptides, the impact of a step-wise increase in negative charge can be monitored. NMR titration experiments with both H3^E3^ and H3^E7^ resulted in clear chemical shift changes for PSIP1^PWWP^ residues that are clustered in and around the aromatic cage (Figure 3a,b and Figure S1). As for H3^A3^, most residues show similar direction of CSPs as for the native peptide, pointing to a similar bound-state conformation (see the trajectory for A51 Figure 3d). Global fits of the NMR titration profiles result in a best-fit K_D_ of 8.5 mM (95% probability limits 6.0–13.1 mM) for binding to the H3^E3^ peptide (Figure 3c). Strikingly, the affinity of H3^E7^ is strongly increased, with a best-fit K_D_ value of 1.2 mM (95% probability limits 1.0-1.5 mM) (Figure 3c). These values correspond to a 2- and 14-fold increase in affinity over the native H3 tail peptide. Comparison of the observed CSPs highlights one residue, PSIP1^PWWP^ K16, with significantly larger shift in the titration with H3^E7^ compared to H3^WT^, H3^A3^ and H3^E3^ (Figure 3d and Figure S1). While in the latter three titrations this residue experiences highly similar CSPs indicating a comparable chemical environment, K16 exhibits a much more pronounced perturbation for its amide proton in the H3^E7^ titration indicating a possible specific intermolecular interaction mediated by this residue.

### 2.3. Dynamic Attractive Electrostatics Drive the Increased Affinity for Glu-Rich H3 Tail Peptides

To see whether PSIP1^PWWP^ K16 could be involved in a specific interaction with the H3^E7^ peptide, we used the NMR-interaction data to obtain a molecular model for the PWWP–H3^E7^ complex using the HADDOCK program [43]. Since the CSPs for the H3^E7^ titration cluster in and around the aromatic cage, we enforced a native-like orientation of the methyllysine and the formation of the intermolecular beta-sheet. Residues in the Glu-rich tail were defined as fully flexible, thus allowing any of these residues to be close to the CSP-identified binding interface of PWWP. The top scoring cluster contained ~86% of all structures calculated, and the top four structures in this cluster are shown in Figure 3e. As a result of the flexible docking protocol, the best-scoring solutions show large variation in orientation of the Glu-rich tail. Nevertheless, most top scoring structures feature a hydrogen-bond between PSIP1^PWWP^ K16 and H3^E7^ E38 (Figure 3e). The latter residue is only present in H3^E7^ while all other peptide models contain the native proline on that position. To test whether the K16-E38 interaction is responsible for the large increase in binding affinity for H3^E7^, we titrated a mutant H3^E7^ peptide retaining the native P38 (H3^PE6^) to PSIP1^PWWP^. Again, the CSPs indicate the peptide binds the expected binding interface in and around the aromatic cage (Figure S1). Notably, the CSP for K16 in PSIP1^PWWP^ is strongly decreased in H3^PE6^ when compared to the H3^E7^ peptide and now very similar to that seen for H3^WT^, H3^A3^ and H3^E3^ that also contain the native P38 in their sequence (Figure 3d). These data thus validate the presence of a specific interaction between K16 and E38 in the complex with H3^E7^ as seen in the structural model. Further support comes from the reduced binding affinity of PSIP1^PWWP^ for the H3^PE6^ compared to the H3^E7^ peptide. Fitting of the NMR titration data resulted in a *K*_D_ of 1.74 mM (95% probability limits 1.4–2.2 mM), which is significantly increased (1.4 fold) over the *K*_D_ for H3^E7^ (1.2 mM) (Figure 3f). Since the relative loss in affinity is less than expected upon removal of a stable hydrogen bond, the K16/E38 interaction may be transient. Furthermore, when compared to the *K*_D_ for H3^E3^ (8.5 mM), it is clear that the K16/E38 interaction is not the sole nor the critical determinant of the affinity gain for H3^E7^ over H3^E3^. Of note, next to K16, the CSP for K14 is increased in both H3^PE6^ and H3^E7^ titrations compared to H3^E3^ (Figure S1), further suggesting that binding of these Glu-rich peptides involves multiple, dynamic electrostatic interactions between the charged patches in both peptide and protein.

### 2.4. Nucleosome-Like Charge Distribution in a Branched H3 Peptide Boosts Affinity 

**Figure 4:**
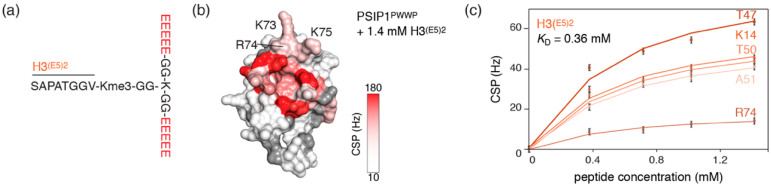
An extended and high-affinity interaction of a branched nucleosome mimicking peptide with PSIP1^PWWP^. (**a**) Sequence of H3^(E5)2^ peptide. (**b**) CSPs upon binding of H3^(E5)2^ plotted and color-coded on the PSIP1^PWWP^ surface, highlighting the extended interaction surface compared to the linear peptides. Peptide concentration is indicated. (**c**) NMR-derived binding curves for H3^(E5)2^ with best fits (solid lines) for selected residues together with best-fit value for the *K*_D_ using a 1:1 binding model global fit of all residues with significant CSP. The plotted binding curves were derived from the CSPs in the ^1^H dimension.

We next sought to improve further on the linear Glu-rich H3 peptides by not only mimicking the abundance of nearby negative charges in the nucleosomal context but also their steric organization. When binding K36me3-modified nucleosomes, PSIP1^PWWP^ engages with both gyres of DNA using two positively charged patches; see Figure 1. The binding interface for the linear H3^E7^ peptide includes K16 that sits in between the two patches, but not the two patches themselves. Therefore, we designed and synthesized a larger and branched peptide structure containing ten glutamic acid residues with the aim of covering the complete nucleosomal DNA binding interface of PSIP1^PWWP^. This peptide (H3^(E5)2^) consists of two negatively charged extensions, each with five glutamate residues, that are connected to the methyllysine containing sequence (Figure 4a). The methyllysine sequence (Kme3-VGGTAPAS) is connected at its N-terminus via two glycine residues to the side chain amino group of a lysine. This lysine forms the branching point and is extended on both its N- and C-terminus by glycine-linked glutamate-rich tails. 

NMR titration experiments with the H3^(E5)2^ peptide showed the expected CSPs for PWWP residues that are in or around the aromatic cage (Figure 4b). In addition, the interaction surface now includes residues K73, R74 and K75 that are part of the native nucleosomal DNA binding interface. These residues were not significantly affected in the previous titration with linear peptides (Figure 2 and Figure 3), indicating that the branched H3^(E5)2^ peptide indeed engages with an expanded surface of PSIP1^PWWP^ that includes at least one of the two positive patches.

**Figure 5:**
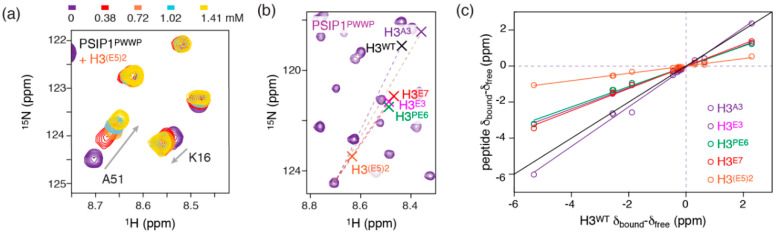
Altered binding mode for Glu-rich peptides binding to PSIP1^PWWP^ (**a**) Zoom on peak trajectories for A51 in the H3^(E5)2^ titration. Color coding indicated. (**b**) Zoom on the ^15^N-HSQC spectrum of unbound PSIP1^PWWP^ with the peak trajectory for A51 and the extrapolated bound-state peak position of A51 schematically indicated for all peptide substrates. (**c**) Comparison of the difference in free and bound chemical shifts (δ_bound_–δ_free_) for binding the H3^WT^ peptide and all peptide-models of this study. Bound-state chemical shifts are extrapolated from the observed CSPs based on the best-fits of the titration data. Black line indicates perfect correlation. Color coding indicated in the figure.

Markedly, upon addition of 1.4 mM peptide, the binding was already close to saturation (Figure 4c, Figure S2 and see also Figure 5a). Correspondingly, a global fit of the NMR-derived binding curves arrives at a *K*_D_ of 360 μM (95% probability limits 190–700 μM). This constitutes a significant improvement in affinity over the linear peptides and a nearly 50-fold increase over the native peptide.

### 2.5. Electrostatic Interactions Alter Aromatic Cage Binding

While the increase in binding affinity for the branched H3^(E5)2^ peptide was expected, analysis of the CSPs in this titration showed a surprising result, as the CSPs observed were much smaller in magnitude than for the linear peptides despite near-saturation of the protein. This can be clearly seen for residue A51, which has the largest CSP in the linear peptide titrations (Figure S1). While for the H3^A3^ titration the ^15^N chemical shift of this residue changed more than 2 ppm (Figure 2a), for the H3^(E5)2^ titration this change is less than 1 ppm (Figure 5a). To analyze this more rigorously, we calculated the chemical shifts of the fully bound state for the A51 backbone amide based on the best-fit of all titration data for all peptide models and plotted this extrapolated bound-state peak position in the spectrum shown in Figure 5b. While the bound-state chemical shifts in the partly neutralized H3^A3^ peptide are close to the shifts observed for the H3^WT^ state, the bound-state shifts are much closer to the unbound state for the Glu-rich linear peptides and even more so for the branched H3^(E5)2^ peptide. This indicates that the chemical environment in the complex is significantly changed upon incorporation of DNA-mimicking negative charges, at least for A51.

Focusing on the residues in and around the aromatic cage, a systematic comparison of the free-bound chemical shift differences in the native peptide complex vs. the differences in derived peptides studied further underscores the observations made for the A51 resonance (Figure 5c). In the complex with the H3^A3^ peptide, the chemical shift differences for the PSIP1^PWWP^ residues are very close to that in the H3^WT^ complex, indicating a similar conformation of the key Kme3–cage interaction and intermolecular β-sheet in the two complexes. Nevertheless, for all three complexes with linear Glu-rich peptides, the bound-state shifts are scaled uniformly to ~55% of their values in the native peptide complex. Moreover, for the H3^(E5)2^ complex the bound-state shifts are scaled to ~20% of their native values.

It is worth emphasizing that the direction of the CSPs that report on trimethyllysine binding, aromatic cage residues M15, W21 and F44, is the same in all peptides, indicative of a conserved binding mode (Figure S4). This also fits with the fact that the Glu-substitutions are all distant from the trimethyllysine. Nevertheless, the uniform scaling of the bound-state shifts strongly suggests a global change in the aromatic cage-trimethyllysine interaction between the native and linear and branched Glu-rich peptides. Since the bound-state shifts are all smaller in the Glu-rich peptides, this indicates that at the point where the protein is saturated with peptide, the trimethyllysine binding pocket is only fractionally occupied. In this scenario, the chemical shifts of the fully bound aromatic cage, δ*_aro,bound_*, are scaled by the fraction in which the cage is bound by the trimethyllysine, *f*_aro,bound_, resulting in the chemical shifts observed at end of the titration with one the peptide substrates, δ*_sat,peptide_*:

δ*_sat,peptide_* = f_aro,bound_*δ*_aro,bound_*, (1)

Notably, it is implicit here that the intrinsic, microscopic affinity for the trimethyllysine interaction is sufficiently low to result in a high dissociation rate and thus fast-exchange between free and bound states. Such low intrinsic affinity is consistent with the low affinity for the native peptide. We exclude the possibility that the trimethylysine is fully bound but in a different manner as we would expect such scenario to result in larger and different CSPs for some residues and smaller CSPs for others, but not a uniform scaling of the CSPs. We thus conclude that increasing binding affinity by incorporating negative charges to mimic the nucleosomal context of H3K36me3 recognition by PSIP1^PWWP^ reduces the aromatic cage occupancy.

## 3. Discussion

**Figure 6:**
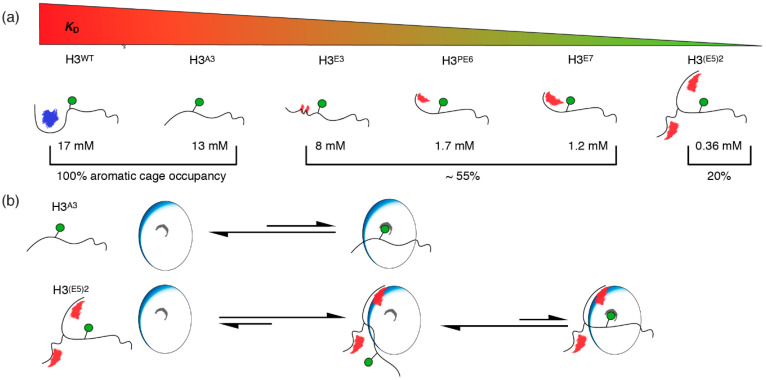
Possible binding mechanism for the studied peptide models and their respective binding affinities. (**a**) A schematic illustration of the peptide models used in this study. A continuous manipulation of the peptide sequence towards the incorporation of increasing negative charges results in a consistent boost of binding affinity (H3^A3^ < H3^E3^ < H3^PE6^ < H3^E7^). This can be further optimized by introducing structural motifs that resemble the electrostatic surrounding of the H3 tail in a nucleosomal context (H3^(E5)2^). However, a decrease in the fraction of occupied aromatic cage suggests that the improved binding is due to electrostatic interactions. (**b**) Possible binding mechanism, explaining the observed effects for mutant peptide binding. H3^WT^ and H3^A3^ engage in a PSIP1^PWWP^ binding that does not include favorable electrostatic interactions and has Kme3 complexation as sole driving force. In case of Glu-rich peptides and especially H3^(E5)2^, the peptide engages in an encounter complex facilitated by electrostatic interactions. Of this complex, only a fraction of Kme3 (20%) is additionally complexed by the aromatic cage (reaction rates indicated with arrow length).

In this work, we explored the potential for peptide-based binders of the PSIP1^PWWP^ domain, a reader of the H3K36me3 modification and involved in HIV integration and cancer. Like other reader domains that specifically interact with trimethylated lysines, PSIP1^PWWP^ features a relatively shallow binding pocket that is generally hard to target for inhibitors. Since the PSIP1^PWWP^ domain binds extremely weakly to H3-tail peptides, but with high affinity to nucleosomes thanks to additional DNA binding, we investigated the potential of mimicking the nucleosomal context into a peptide model. From a series of NMR titration experiments with trimethyllysine peptides (H3^A3^, H3^E3^, H3^PE6^, and H3^E7^), we found that binding affinity indeed increases in stepwise manner with electronegativity (Figure 6a). For the best linear peptide, containing a Glu-rich tail of seven residues, affinity was increased ~ten-fold over the native peptide. We additionally designed a branched peptide H3^(E5)2^ with two Glu-rich tails to mimic the structural arrangement of the negative charges over two DNA helices in the nucleosomal context. The affinity of this peptide for PSIP1^PWWP^ was in the high micromolar range, a five-fold increase compared to the best linear model, and a ~50-fold improvement over the native H3-tail peptide, yet still roughly 50–100× weaker than the binding to K36me3-modified nucleosomes.

For the two highest affinity peptides, H3^E7^ and H3^(E5)2^, the involvement of positively charged residues from the DNA binding surface of PSIP1^PWWP^ could be established from the NMR data. In the H3^E7^ complex, we could identify an involvement of K16, while in the branched H3^(E5)2^ complex, the patch around R74 is part of the binding interface. In the native nucleosomal context, a patch around K16, a patch around R74 and a patch around K39 are responsible for the tight association with H3K36me3 nucleosomes. The peptide models, including the branched peptide, thus seem to engage only part of the available binding surface on PSIP1^PWWP^, leaving room for further improvement.

The interactions between the Glu-tails and the positive patches in PSIP1^PWWP^ are likely highly dynamic electrostatic interaction rather than well-defined side chain interactions. This is shown in the mild loss in affinity upon removal of a specific Glu-Lys interaction (E38-K16) and the overall limited size of CSPs for the positively charged residues in PSIP1^PWWP^. It is also known that electrostatic interaction interfaces can remain solvated and therefore do not cause large changes in the chemical environment of the backbone amides [44,45].

While the introduced negative charges boost binding affinity as intended, the trimethyllysine–aromatic cage interaction seemed to be reduced. Mechanistically, we interpret this as an altered balance in a two-step binding process, as shown in Figure 6b. The charged peptides first bind electrostatically using their Glu-rich tail, after which the trimethyllysine can dock into the aromatic cage. Our peptides boosted affinity by stabilizing primarily the first complexed state, akin to encounter complex [46], and not the second complexed state with the trimethyllysine also engaged. We thus find that the electrostatic interactions compete with effective binding of the trimethyllysine, “pulling” it out of the aromatic cage, resulting in decreased occupancy of the aromatic cage as evidenced by the NMR chemical shifts.

The molecular reason for the “pulling” effect of the Glu-rich tails is not fully clear. We note that the docking models of the H3^E7^ complex do not suggest some sort of steric incompatibility in simultaneous binding of the aromatic cage and the positive patches of PSIP1^PWWP^. This would indicate that the electrostatic interactions are most favorable when the peptide is “pulled” further away from the aromatic cage. The lack of large CSPs on the positive patches distal to the aromatic cage would indicate that these interactions are highly dynamic as expected in encounter states. 

Interestingly, our data indicate the aromatic cage is bound to a similar extent (~55%) for all three linear Glu-rich peptides, while for double-tailed branched peptide, the occupancy drops to ~20%. This indicates that, in terms of the incompatibility with aromatic cage binding, the number of charges is not decisive. We speculate that the decreased cage occupancy for the H3^(E5)2^ peptide is due to the highly flexible nature of peptide, featuring three double glycine linkers between the trimethyllysine part and the Glu-rich tails. This design maximizes conformational freedom and effectively decouples cage binding from the Glu-tail interaction. As a result, the linkage effect [47] is reduced, and the inherently weakly bound trimethyllysine can more readily dissociate from the aromatic cage. 

Alternatively, one could imagine that the positively charged trimethyllysine can transiently interact with the Glu-tails of the peptide. This would mean that the aromatic cage and the Glu-rich tail are in competition for binding the trimethyllysine, which could also explain the reduced cage occupancy. Within the native nucleosomal context, the negative charges are fixed in the DNA while the H3K36me3 site has limited conformational freedom, being close to the entry point of the H3 tail in between the DNA gyre (see Figure 1). We have highlighted before the remarkable match between the nucleosomal context of the H3K36me3 site and the PSIP1^PWWP^ [30]; the current observations underscore the challenges in mimicking this environment in peptide-based systems. 

For other K36me3 reader domains, which generally feature a DNA binding surface directly adjacent to the aromatic cage [25,36], a similar strategy to engineer potent inhibitors may face the same challenges to capture both aromatic cage and DNA binding patches in one molecule. Nevertheless, it should be noted that PSIP1PWWP has extremely low affinity for the H3K36me3 peptide and thus presents a worst-case scenario that in hindsight will particularly sensitive to a “pulling” effect as observed here. For other reader domains, such as the CBX8 chromodomain [48], the DNA binding surface may be more distant from the aromatic cage making the strategy to translate the multivalent chromatin association into a single compound less suitable. Of note, instead of blocking the cage and DNA interaction, it would be worthwhile to block the aromatic cage but enhance DNA binding. An inhibitor of CBX7 chromodomain was found to enhance DNA binding by likely stabilizing a DNA-binding competent conformation of the protein [24]. In cells, this led to an effective relocation of the target protein on chromatin away from the its native sites. A similar strategy could also be conceived for PSIP1PWWP, although comparison of free and bound state PWWP structures does not suggest that there are specific conformational changes involved in DNA binding [36].

To improve the potency of the peptides identified in the current framework, the following points can be considered: (i) increase the intrinsic, microscopic affinity of the aromatic cage targeting part; (ii) reduce the flexibility between the DNA-mimicking and aromatic cage targeting part, making sure the proper steric fit to the protein surface is maintained; and (iii) expand the DNA-mimicking part to engage more of the DNA-binding surface. For (i) optimization of the sequence, derivatization of the Kme3 group and inclusion of non-natural groups could all be used, as reviewed in [49]. Cyclization of the peptide could be used to constrain the conformational freedom within the molecule, as well as a means to engage more of the DNA binding surface.

Since the lack of enzymatic activity for reader domains such as the PWWP domain means that screening and selection of compounds for reader domains is firstly based on affinity measurements, our case further highlights the need to carefully validate the binding mode. With an only partially occupied aromatic cage, our best binding peptide will be much less effective at inhibiting PSIP1^PWWP^ than would have been expected from the affinity.

## 4. Materials and Methods 

### 4.1. Protein Expression

For NMR studies, PSIP1^PWWP^ (AA 2-100, Uniprot-ID: O75475) was expressed as a GST-fusion protein in BL21 Rosetta2 (DE3) cells (Novagen). PSIP1^PWWP^ was produced in M9 minimal medium containing ^15^NH_4_Cl. Cells were grown to an OD of 0.6 and induced with 500 μM IPTG. The protein was expressed for 3 h at 37 °C. The cells were harvested in lysis buffer (50 mM Tris pH 8.0, 300 mM KCl, 0.5 mM PMSF, 1 mM DTT, 3.3 mM TritonX-100, complete EDTA-free protease inhibitor (Roche, Penzberg, Germany) and lysed by French-press. The soluble fraction was collected after ultracentrifugation at 25,000 rpm and 4 °C for 40 min, and subsequently loaded on a 5 mL GSTrap HP column (GE Healthcare, New York, NY, USA). The column was washed with wash buffer I (50 mM Tris pH 8.0, 300 mM KCl, 1 mM DTT, complete EDTA-free protease inhibitor (Roche)) and wash buffer II (50 mM KPi pH 7.0, 100 mM KCl, 1 mM DTT, complete EDTA-free protease inhibitor (Roche)). The GST-fusion protein was eluted using elution buffer (50 mM KPi pH 7.0, 100 mM KCl, 50 mM reduced glutathione, 1 mM DTT, complete EDTA-free protease inhibitor (Roche)). Thrombin cleavage of the GST-tag was carried out for 3 h at 37 °C after adding 10U of thrombin to the combined eluted fractions, then quenched by addition of 1 mM PMSF and incubation for 15 min at 37 °C. The PSIP1^PWWP^ domain was then purified using ion-exchange chromatography over a HiTrap SP HP column (GE Healthcare). A gradient from IEX buffer (20 mM KPi pH 7.0, 0.2 mM PMSF, 0.5 mM DTT) w/o KCl to IEX buffer with 1 M KCl was used to elute PSIP1^PWWP^ from the column. The PWWP-containing fractions were then combined and dialyzed to NMR buffer (20 mM NaPi (pH = 7.0), 150 mM NaCl, cOmplete protease inhibitor, 10% (*v/v*) D_2_O). 

### 4.2. Peptides

The H3 tail peptides H3^A3^ (Ac-SAPATGGV[Kme_3_]KPAAYAPG-NH_2_), H3^E3^ (Ac-SAPATGGV[Kme_3_]KPEEYEPG-NH_2_), H3^E7^ (Ac-SAPATGGV[Kme_3_]KEEEEEEE-NH_2_) and H3^PE6^ (Ac-SAPATGGV[Kme_3_]KPEEEEEE-NH_2_) were supplied by Biomatik (Kitchener, Canada) with a > 95% purity as TFA salt. All peptides were dissolved in NMR buffer (20 mM NaPi (pH = 7.0), 150 mM NaCl, cOmplete protease inhibitor, 10% *(v/v*) D_2_O). The pH for all peptide stocks was adjusted to 7.0 using NH_4_OH. Determination of peptide stock concentrations was done using 1D NMR spectra with TSP as reference compound. The integral of the isolated singlet arising from TSP methyl groups (0.0 ppm) was put in relation to the peak for Val35 methyl groups in the peptides (~0.8 ppm) to back-calculate the effective peptide concentrations. The Val signal was verified by 2D NMR spectra (TOCSY, ^13^C-HSQC). The stock concentrations were 70.54 mM (H3^A3^), 50.98 mM (H3^E3^), 29.74 mM (H3^E7^), 11.85 mM (H3^(E5)2^) and 26.8 mM (H3^PE6^).

### 4.3. Synthesis H3^(E5)2^

H3^(E5)2^ was synthesized manually, using Fmoc-based solid phase peptide synthesis. The backbone sequence (EEEEEGGKGGEEEEE) was synthesized on a Rink amine resin (Rapp polymer TentaGel S RAM) with the lysine side chain amine protected with an Mmt group. Deprotection was carried out for 5 min with 40% piperidine in DMF, then 15 min with 20% piperidine in DMF, followed by three washes with DMF. The coupling steps were performed as double couplings for 40 min each with 4 eq. amino acid together with HBTU/HOBt activation and DIPEA as base. After every coupling step and at the end of the synthesis, free backbone amine was capped by acetylation. For branching, Lys(Mmt) was selectively deprotected on-resin with a cleavage solution of 2% (*v/v*) TFA, 98% (*v/v*) DCM. Completion was ensured by monitoring the yellow color of the trityl-cation. The synthesis of the branch was propagated via the ε-N of the Lys side chain. All following amino acids were coupled using the DIC/HOAt strategy with 10 eq. amino acid and double coupling and coupling time of 1 hr. The crude product was cleaved off the solid support using a cleavage solution of 95% (*v/v*) TFA, 2.5% (*v/v*) TIPS and 2.5% (*v/v*) H_2_O. Purification of the peptide was done using LC-MS, isolating a species with *m*/*z* peaks at 1349.8 and 1350.2, both slightly (1.6 Da) off from the expected monoisotopic mass (1348.1) and most abundant mass (1348.6) peaks for [M + H]^2+^ ion from the peptide with exact mass 2696.15, likely due to a calibration error. NMR analysis further confirmed the identity of the purified product. The product was dissolved in H_2_O and evaporated three times to co-evaporate residual TFA. Afterwards, the product was lyophilized and dissolved in NMR buffer. The stock concentration was found to be 11.85 mM at pH 6.97 using the method described above.

### 4.4. NMR Spectroscopy

All NMR experiments were carried out at 298 K on a Bruker Avance spectrometer operating at 750 MHz ^1^H Larmor frequency with room-temperature probe. Processing was done using the Bruker’s TopSpin. Spectra were analyzed using Sparky (Goddard and Kneller, UCSF). Samples for the titration experiments contained ~300 µM ^15^N-PSIP1^PWWP^ in NMR buffer (20 mM NaPi, pH 7.0, 150 mM NaCl, 10% D_2_O, cOmplete EDTA-free Protease Inhibitor Cocktail (Roche)). 

### 4.5. Titration Data Analysis

Titration data were fitted using MatLAB scripts (available upon request) using the fast-exchange assumption [50]. Briefly, titration profiles from non-overlapping residues with CSPs larger than 50 Hz (H3^A3^, H3^E7^, H3^PE6^), 20 Hz (H3^E3^) or 10 Hz (H3^(E5)2^) were selected to be included in a simultaneous fit of titration curves to determine the *K*_D_. Profiles were selected either form the ^1^H or ^15^N dimension. A 1:1 binding model was assumed. Fitting parameters are residue-specific values for an offset term to compensate for inaccuracies in the initial peak position (in all cases <10 Hz), the residue-specific values for the free-bound chemical shift differences, and the global *K*_D_ value.

(2) $$\chi^{2}=\overset{M}{\underset{j}{\sum }}\overset{N}{\underset{i}{\sum }}{\left(\frac{\delta_{\mathrm{calc}}\left(K_{D},\mathrm{offset}\right)-\delta_{obs}}{\sigma_{\mathrm{nucleus}}}\right)}^{2}$$

where δ is the peak position, *nucleus* = ^15^N or ^1^H, σ_1H_ = 1.5 Hz and σ_15N_ =1.5 Hz, *i* runs over all *N* titration points and *j* runs over all *M* selected resonances. The degrees of freedom are equal to total number of points *NM* compensated for the number of global variables (K_D_), the *M* offset-variables and *M* chemical shift differences. To obtain the 95% confidence limits in the K_D_, a one-dimensional grid search was performed either systematically increasing or decreasing the *K*_D_ until the reduced χ^2^ exceeded the 95% critical value according to an *F*-test.

Analysis of the bound-state chemical shifts was based on selected residues K14 (^1^H/^15^N), M15 (^1^H), W21 (^1^H), F44 (^15^N) T47 (^1^H/^15^N), H48 (^1^H), T50 (^1^H/^15^N) and A51 (^1^H/^15^N) due to their significant shift in all experiments.

### 4.6. Data-Driven Docking

The structural model of the PSIP1^PWWP^/H3^E7^ complexes was determined using the experimental NMR data in the data-driven docking software HADDOCK [43]. The H3^WT^ structure [30] was used as a template to build an atomic model for the H3^E7^ peptide including amidation (C-terminus) and acetylation (*N*-terminus). This was docked to the PSIP1^PWWP^ surface from the nucleosome-bound structural model [36]. Insertion into the aromatic cage was enforced by unambiguous restraints between K36me3 and PSIP1^PWWP^ residues M15, Y18, W21 and F44. Kme3 was set was an active residue. The H3 tail sequence part of the peptide was directed into a native confirmation by enforcing O-N hydrogen bonds between PSIP1^PWWP^ T50(O) and K36me3 (N) as well as T50 (N) and G35 (O). Glutamate residues on the peptide were defined as fully flexible segment. A total of 13 ambiguous restraints were included for PSIP1^PWWP^ residues with observed CSP and neighboring solvent accessible residues (see Figure 3b and Figure S3). Docking was done with increased MD steps for rigid body high temperature TAD (2000), first rigid body cooling stage (2000), second cooling stage with flexible side-chains at interface (4000) and third cooling stage with fully flexible interface (4000) [51] Of the 200 water-refined solutions, 198 were clustered in four clusters (Figure S3).

## 5. Conclusions

In this work, we presented rationally designed peptide-based binders of a reader of the H3K36me3 modification, the PWWP domain of transcriptional co-regulator PSIP1. Inclusion of negative charges to mimic the nucleosomal context of the native binding mode resulted in a ~50-fold improvement in binding affinity over a simple histone peptide. The introduced charges stabilize an encounter complex, resulting in decreased trimethyllysine binding. We conclude that combined targeting of both the electrostatic surface and the shallow Kme3 binding epitope of K36me readers may be a promising strategy in the development towards potent chemical probes and inhibitory compounds, but it needs careful optimization and validation of both binding modes.

## Supplementary Materials

The following are available online. Figure S1: Chemical shift perturbations for all H3K36me3 peptides. Figure S2: Titration data fits for all H3K36me3 peptides. Figure S3: Docking results for the H3E7 complex. Figure S4: Overlay of all peptide-added PSIP1^PWWP^ spectra.

## Notes

**Sample Availability:** Not available.
