# Supplementary material for: Mimicking the Nucleosomal Context in Peptide-Based Binders of a H3K36me Reader Increases Binding Affinity While Altering the Binding Mode
Source: Molecules. 2020 Oct 26;25(21):4951. doi: 10.3390/molecules25214951 (PMC7662849; doi:10.3390/molecules25214951)
Supplement: Supplementary file 1 [file molecules-25-04951-s001.pdf]

Supplementary Materials

Mimicking the nucleosomal context in peptide-based binders of a H3K36me reader increases binding affinity while altering the binding mode

Velten Horn [1], Seino A.K. Jongkees [2] and Hugo van Ingen [1,3,*]

[1] Macromolecular Biochemistry, Leiden Institute of Chemistry, Leiden University; v.horn@lic.leidenuniv.nl
[2] Chemical Biology and Drug Discovery, Utrecht University; s.a.k.jongkees@uu.nl
[3] NMR Group, Bijvoet Centre for Biomolecular Research, Utrecht University; h.vaningen@uu.nl
* Correspondence: h.vaningen@uu.nl; Tel.: +31-30-253 9934

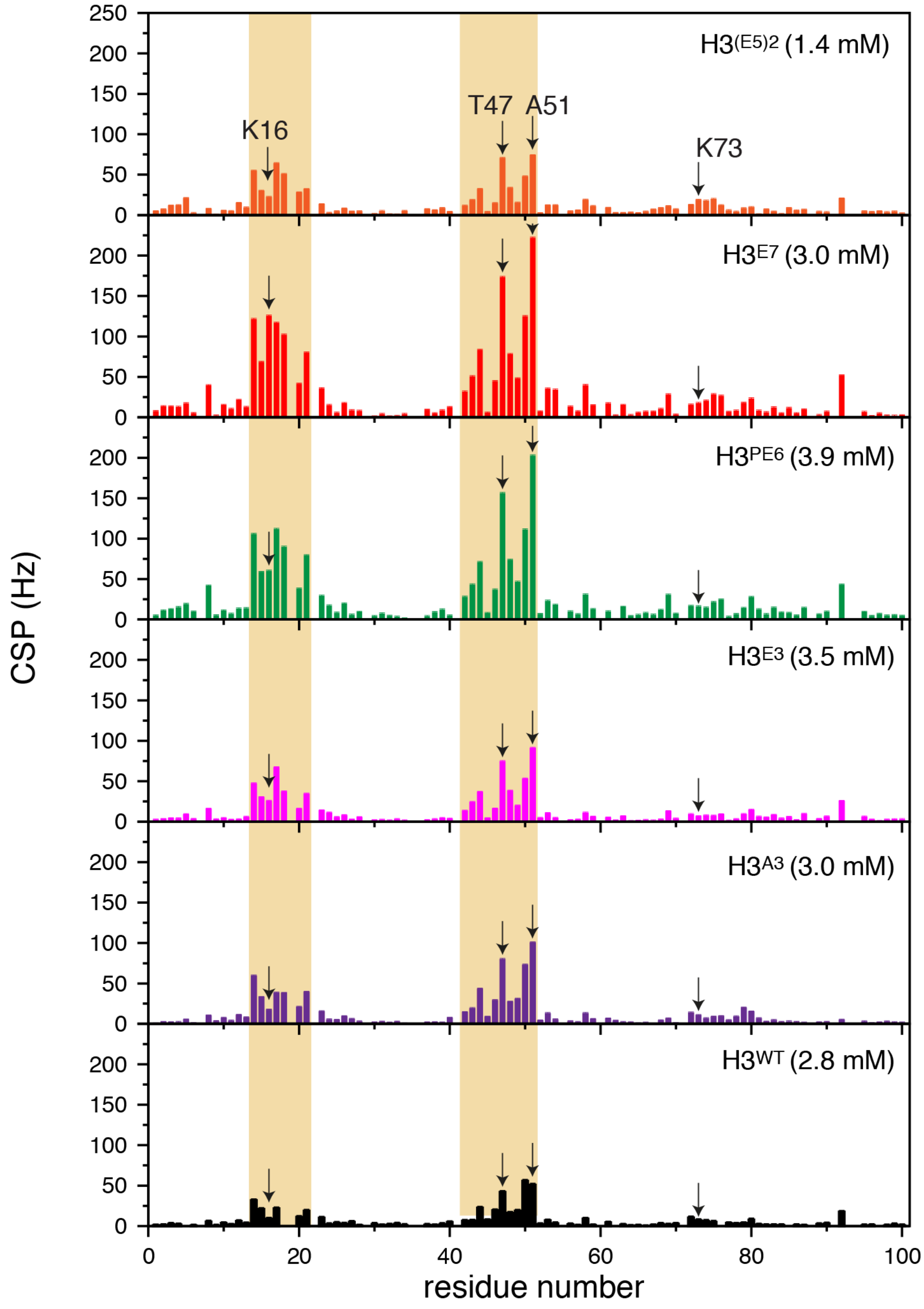

Figure S1. Chemical shift perturbations (CSPs) for all H3 peptide models in this study at indicated peptide concentrations. Except for H3(E5)2 where the maximum peptide concentration was used, all concentrations were chosen around 3 mM such that not only the location of affected residues but also the relative size of the CSPs and thus binding affinity can be compared. Two main regions of CSPs are conserved in all peptides (highlighted in yellow) corresponding to the PWWP surface covering and around the aromatic cage. Key residues in the analysis are indicated. H3^{WT} data from ref. [30] shown for comparison. CPSs are calculated as the 2D peak displacement expressed in Hz, i.e. using a weighting factor of 10 for the 15N CSPs when expressed in ppm's.

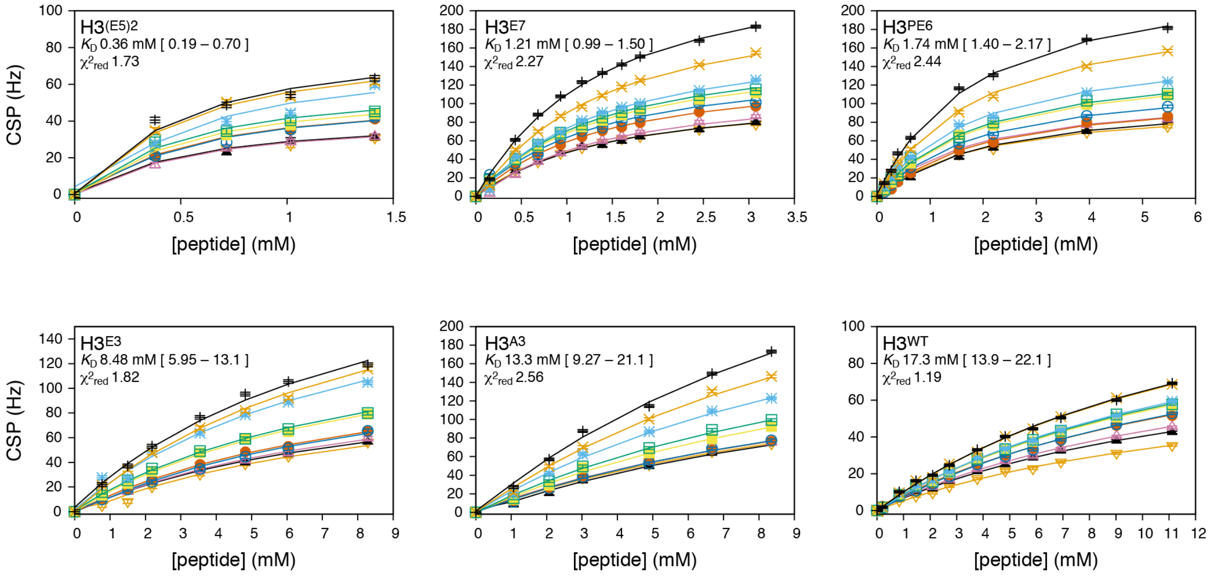

Figure S2 Global fits of NMR titration curves to 1:1 binding model for all H3 peptide models in this study. The ten curves with largest CSP are shown in each case, color coding is based on size of CSP, not on residue. CSP profiles were taken from either the ^{1}H or ^{15}N dimension (see Materials and Methods). Best fit value of the K_D is given together with F-test based 95% confidence interval limiting values in brackets. The χ^2_{red} of the best-fit is also reported. H3^{WT} data from ref. [30] shown for comparison.

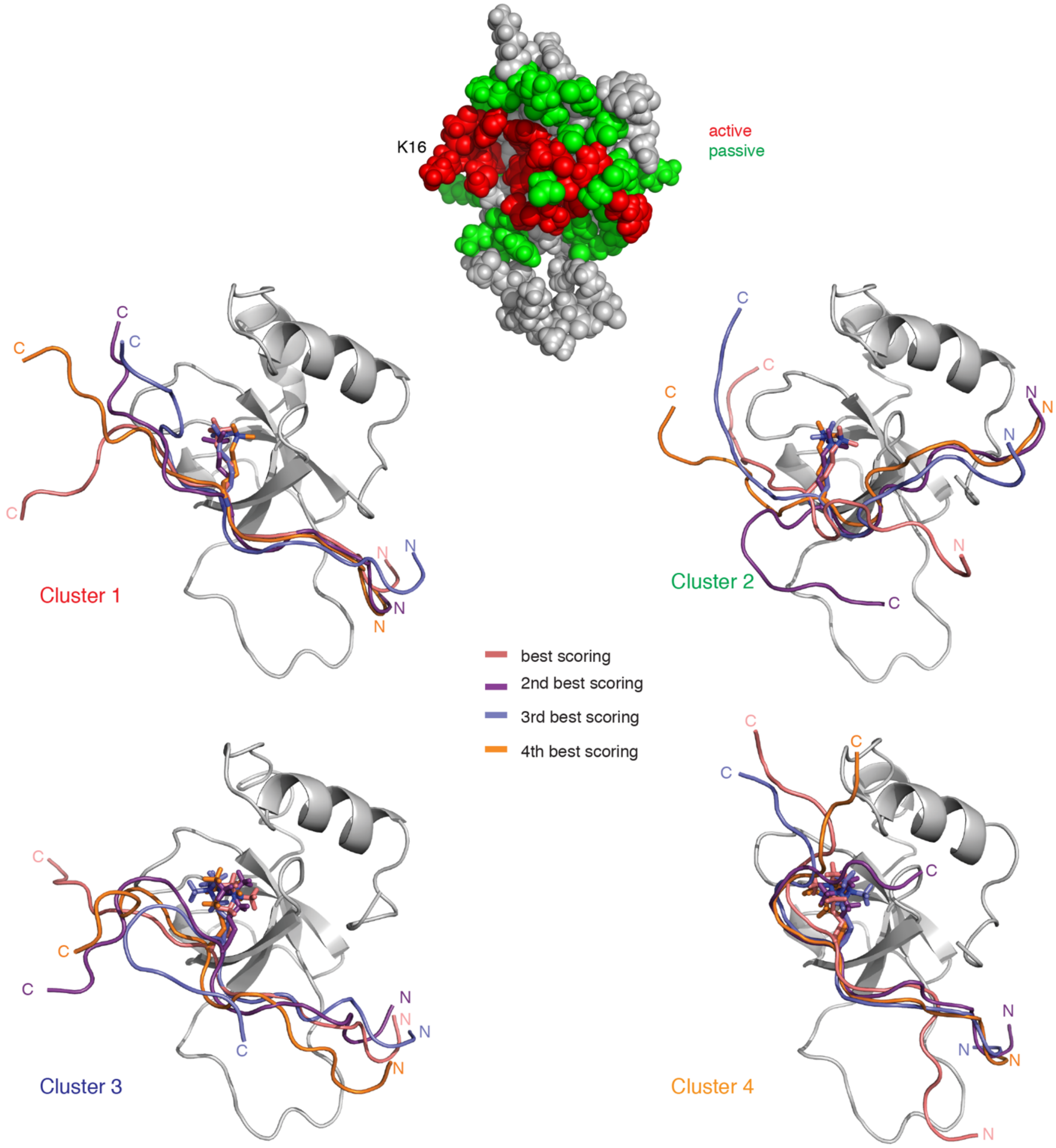

	Cluster 1	Cluster 4	Cluster 2	Cluster 3
HADDOCK score	-124.2 +/- 4.4	-116.5 +/- 10.2	-112.7 +/- 11.8	-80.5 +/- 14.6
Cluster size	172	5	14	7
RMSD from the overall lowest-energy structure	5.4 +/- 0.5	6.2 +/- 0.3	2.9 +/- 2.0	5.5 +/- 0.6
Van der Waals energy	-57.0 +/- 9.8	-64.6 +/- 3.9	-47.7 +/- 6.1	-40.3 +/- 6.8
Electrostatic energy	-373.0 +/- 60.2	-327.3 +/- 73.8	-413.1 +/- 115.1	-243.0 +/- 35.6
Desolvation energy	1.9 +/- 4.3	6.3 +/- 4.5	10.3 +/- 8.9	1.2 +/- 7.8
Restraints violation energy	55.3 +/- 32.62	72.4 +/- 52.17	72.8 +/- 35.88	72.3 +/- 30.06
Buried Surface Area	1568.6 +/- 97.8	1764.7 +/- 84.7	1507.2 +/- 67.6	1231.0 +/- 174.4
Z-Score	-0.9	-0.5	-0.3	1.7

Figure S3 Docking results for H3E7 and PSIP1-PWWP **(top)** Active residues (red) and surrounding surface accessible passive residues (green) used as input for HADDOCK modelling (**middle**) Cartoon view of all obtained clusters of docking solutions, showing for each cluster the top four structures. K36me3 is displayed as sticks. C-terminus (poly-Glu) and N-terminus (native H3 sequence) are indicated. (**bottom**) Docking statistics are shown for all clusters in their scoring order.

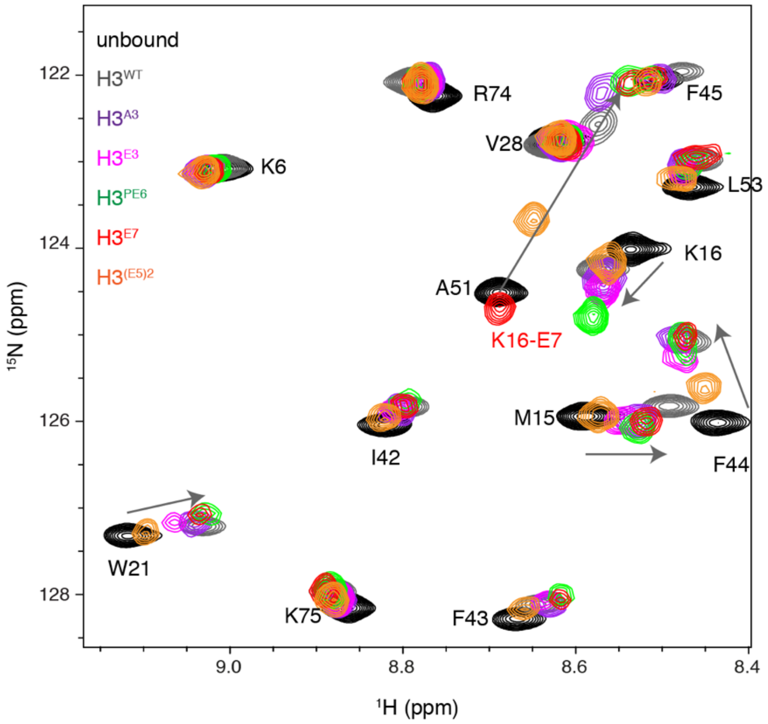

Figure S4 NMR spectra of last titration points for all H3 peptide models in this study overlaid, showing conservation of the direction of chemical shift perturbations for trimethyllysine binding reporter residues . $H3^{WT}$ data from ref. [30] shown for comparison.
